# MicroRNA expression in benign breast tissue and risk of subsequent invasive breast cancer

**DOI:** 10.1371/journal.pone.0191814

**Published:** 2018-02-12

**Authors:** Thomas Rohan, Kenny Ye, Yihong Wang, Andrew G. Glass, Mindy Ginsberg, Olivier Loudig

**Affiliations:** 1 Department of Epidemiology and Population Health, Albert Einstein College of Medicine, Bronx, New York, United States of America; 2 Warren Alpert Medical School of Brown University, Providence, Rhode Island, United States of America; 3 Center for Health Research, Kaiser Permanente Northwest, Portland, Oregon, United States of America; 4 Hackensack University Medical Center, Hackensack, New Jersey, United States of America; University of South Alabama Mitchell Cancer Institute, UNITED STATES

## Abstract

MicroRNAs are endogenous, small non-coding RNAs that control gene expression by directing their target mRNAs for degradation and/or posttranscriptional repression. Abnormal expression of microRNAs is thought to contribute to the development and progression of cancer. A history of benign breast disease (BBD) is associated with increased risk of subsequent breast cancer. However, no large-scale study has examined the association between microRNA expression in BBD tissue and risk of subsequent invasive breast cancer (IBC). We conducted discovery and validation case-control studies nested in a cohort of 15,395 women diagnosed with BBD in a large health plan between 1971 and 2006 and followed to mid-2015. Cases were women with BBD who developed subsequent IBC; controls were matched 1:1 to cases on age, age at diagnosis of BBD, and duration of plan membership. The discovery stage (316 case-control pairs) entailed use of the Illumina MicroRNA Expression Profiling Assay (in duplicate) to identify breast cancer-associated microRNAs. MicroRNAs identified at this stage were ranked by the strength of the correlation between Illumina array and quantitative PCR results for 15 case-control pairs. The top ranked 14 microRNAs entered the validation stage (165 case-control pairs) which was conducted using quantitative PCR (in triplicate). In both stages, linear regression was used to evaluate the association between the mean expression level of each microRNA (response variable) and case-control status (independent variable); paired t-tests were also used in the validation stage. None of the 14 validation stage microRNAs was associated with breast cancer risk. The results of this study suggest that microRNA expression in benign breast tissue does not influence the risk of subsequent IBC.

## Introduction

Elucidation of the molecular basis of breast cancer development and progression has the potential to lead to new approaches to prevention of this disease and to improvements in its prognosis. With respect to prognosis, mRNA-based transcriptional profiling of breast cancer tissue has been shown to predict clinical outcome [1] and to perform better than current clinical criteria [2]. Insight into the molecular basis of breast cancer development might come from the transcriptional profiling of benign breast disease (BBD) tissue, given the increased breast cancer risk associated with BBD [3]. For this purpose, because of the impracticality of collecting fresh tissue from large numbers of individuals, there is a need for prospective cohort investigations involving study subjects for whom archival formalin-fixed, paraffin-embedded (FFPE) tissue is available.

MicroRNAs (miRNAs) are a class of endogenous, small (19–25 nucleotides) non-coding RNAs that control gene expression by directing their target mRNAs for degradation and/or posttranscriptional repression [4]. Abnormal expression of miRNAs is thought to contribute to the development and progression of breast and other cancers [4–6], and recent clinical studies have begun to evaluate miRNA expression profiling of tumors as potential prognostic tools [7]. Our data and those of others [8–10] have shown that miRNA expression profiling of FFPE archival tissue is feasible, but we are not aware of any large-scale studies in humans that have prospectively examined miRNA expression in FFPE tissue in relation to the risk of cancer development. Therefore, we conducted two case-control studies, the first to identify and the second to validate the association between expression of miRNAs in BBD tissue and risk of subsequent invasive breast cancer. The studies were nested in a large, epidemiologically well-characterized cohort of women who had a biopsy for BBD, for whom archival FFPE breast tissue was accessible, and who were followed up to determine the occurrence of breast cancer subsequent to the BBD biopsy.

## Patients and methods

### Study population

We conducted our investigation by performing case-control studies nested within a cohort of women whose biopsies for benign breast disease (BBD) were performed within the Kaiser Permanente Northwest Region (KPNW) health care system. KPNW is a prepaid health plan that provides comprehensive medical care for over 480,000 members in facilities located in Southwest Washington and Northwest Oregon.

The study was retrospective, entailing use of routinely collected data and archival benign breast disease tissue. Hence, no verbal or written consent was obtained. All data were fully anonymized before access by the researchers, and the study was approved by the Committee on Clinical Investigations of the Albert Einstein College of Medicine and the Kaiser Permanente Northwest Biospecimen Review Committee.

### Cohort definition

The cohort consisted of the 15,395 women aged 21 to 85 years who received a histopathologic diagnosis of BBD at KPNW between August 3, 1971 and December 31, 2006. Women who were diagnosed with invasive breast cancer prior to or within one year of their first biopsy for BBD were excluded from the cohort. Women in the cohort were followed until they developed breast cancer, died, left the KPNW Health Plan, or until July 1, 2015, the end of follow-up of the cohort, whichever came first.

### Case-control design and selection of study subjects

We conducted two case-control studies nested within the BBD cohort. The first case-control study was used to identify miRNAs potentially associated with altered risk of subsequent invasive breast cancer (discovery stage), and, following an intermediate validation step, the second case-control study (validation stage), nested within the same cohort, was used to validate the association for the 14 miRNAs both most strongly associated with altered risk in the discovery stage and most strongly correlated with quantitative PCR measures of miRNA expression in the intermediate stage (see below).

#### Case definition, ascertainment, and selection

Cases were women with a biopsy for BBD and with a subsequent first diagnosis of invasive breast cancer (at least one year after the index BBD biopsy). The occurrence of breast cancer in the cohort was ascertained by linking records from the BBD cohort to the KPNW Tumor Registry. The KPNW Tumor Registry has operated continuously since 1960 and is approved by the Commission on Cancer of the American College of Surgeons. It was computerized in 1978 and includes all cases diagnosed since 1960. Cases of cancer are identified primarily from pathology reports forwarded directly from the KPNW Department of Pathology to the Tumor Registry at the time of diagnosis. These cases are supplemented by examination of the inpatient discharge logs, referrals to radiation oncologists, and surveillance of radiology reports marked as particularly suspicious for cancer. The terms of insurance coverage by Kaiser Permanente serve to concentrate members’ medical care within the walls of KPNW, particularly for chronic diseases such as cancer. (In a review of 1,846 incident breast cancers diagnosed during the period 1960-1985, it was found that 99.1% were diagnosed and treated at KPNW, 0.6% were diagnosed at KPNW and treated elsewhere, 0.1% were diagnosed at autopsy, and only 0.2% were diagnosed elsewhere but treated at KPNW (A.Glass, personal communication).) The KPNW Tumor Registry has an excellent follow-up rate, even for women who are no longer health plan members, and it has maintained a follow-up rate of 98% of patients (living and dead) enrolled since its reference date of 1960.

Our goal was to include in the discovery case-control study approximately 2/3 of the total number of cases expected to be ascertained in the cohort through the end of the study period. S1 Fig shows that, after exclusions for various reasons, the discovery stage included 316 cases (and matched controls) and the test stage included 165 cases (and matched controls).

#### Control definition and selection

Controls were women with a biopsy for BBD who were alive but had not developed breast cancer during the same follow-up period as that for the corresponding cases. For each of the cases, we randomly selected one control using risk-set sampling. Each control was individually matched to the corresponding case on age (+/- 1 year) and age at diagnosis of BBD (+/- 1 year)(and implicitly, given the risk-set sampling, on duration of membership of KPNW), and was sampled randomly from the risk-set with replacement [11]. In addition to being alive and free of invasive breast cancer, the controls were required not to have undergone a mastectomy before the date of diagnosis of breast cancer for its matched case.

### Clinical/Epidemiological data

KPNW uses unique and permanent health record numbers to identify all members. All encounters with the medical care system—clinic visit, prescription, operation, laboratory test, and so on—are linked by this identification number and are recorded in the single health record. For the present study, risk factor information was obtained by abstracting data from the KPNW medical records using a standardized chart abstraction protocol. This yielded information on: age at menarche; age at first live birth; number of pregnancies; menopausal status; family history of breast cancer in a first degree relative; height; weight; ever use and duration of use of cigarettes and number of cigarettes smoked per day; ever use and duration of use of hormone replacement therapy; and history of and date of bilateral oophorectomy and hysterectomy. In addition, for the breast cancer cases, we obtained information from the KPNW Tumor Registry on the date of diagnosis and tumor laterality.

### Tissue acquisition

The KPNW Department of Pathology has retained slides of all its cases since 1970. The files of slides are complete and are easily accessed. Blocks are similarly available and filed in the KPNW warehouse adjacent to the pathology laboratory. For those subjects selected for inclusion in the study described here, attempts were made to retrieve tissue blocks from the archives.

### Histopathology

Histological sections from the BBD tissue blocks were reviewed and classified according to the well-established criteria of Page and colleagues [12–15] into the following categories: non-proliferative lesion; proliferative disease without atypia; and atypical hyperplasia (ductal and/or lobular). Proliferative disease was considered to be present if any of the following changes were observed: ductal hyperplasia, papilloma, radial scar, or sclerosing adenosis. Cysts, aopcrine metaplasia, fibroadenoma without epithelial hyperplasia, or columnar cell change were considered to be non-proliferative unless they contained one of the listed proliferative lesions. The histologic sections were reviewed by the study pathologist without knowledge of the case-control status of the study subjects.

### Laboratory assays

The study was conducted in several stages. In the first (discovery) stage, we analysed, in duplicate, RNA extracted from the FFPE BBD tissue of 316 cases and their matched controls using the Illumina MicroRNA Expression Profiling Assay to identify breast cancer-associated miRNAs. In an intermediate stage, the 71 miRNAs that were most differentially expressed between the cases and controls, together with 10 miRNAs associated with atypical hyperplasia, were further evaluated using quantitative PCR (qPCR) in 30 BBD tissue samples (15 case-control pairs). The 14 miRNAs that showed the strongest correlations between the qPCR and the Illumina results and that were most strongly associated with breast cancer risk were carried through to the validation stage in which qPCR was used to assay expression levels of these miRNAs in 165 case-control pairs. Assays were conducted in batches consisting of cases and their matched controls but without knowledge of case-control status.

#### RNA extraction

For each study subject, a minimum of 6 morphologically guided 1mm tissue cores was obtained using a chemicon microarrayer from the identified benign lesions and collected in RNase-free eppendorf tubes. For RNA extraction, we used the method that we have described elsewhere, and that has been shown to yield very high correlations (r>0.94) between global miRNA expression profiles obtained using the Illumina miRNA expression profiling platform on RNA extracted from matched fresh and FFPE tissue [16]. In brief, the FFPE tissue cores were washed, rehydrated and homogenized in the presence of proteinase K digesting buffer (50mM Tris-HCl pH7.5, 75mM NaCl, 5mM Cacl2 and 0.1% SDS) and 30mg/ml proteinase K (Roche Diagnostics, Inc.). After Butanol-1 extractions, the 150μl tissue digests were subjected to TRIzol (Invitrogen) extractions, following the manufacturer’s instructions. The RNA obtained from the upper layer was precipitated at -20°C O/N and centrifuged to obtain RNA pellets. The RNA pellets were washed, resuspended in 20μl in 1x Tris HCl pH7.5, EDTA and incubated at 70°C for 30min for removal of cross-linkages. The RNA was then quantified on a Nanodrop ND-1000 and on an Agilent Bioanalyzer Nano chip before being stored at -80°C.

#### Discovery stage—MiRNA expression profiling

For the first case-control study, miRNA expression profiling of the FFPE specimens was performed using the Illumina MiRNA Expression Profiling Assay with 200ng of total FFPE RNA. This assay, now discontinued, was an adaptation of the DASL^®^ (cDNA-mediated Annealing, Selection, Extension, and Ligation) Assay. With this assay, we simultaneously analyzed the expression of 1,145 miRNAs from FFPE RNA specimens in batches of 48 individual samples (24 matched case-control pairs), in duplicate (96 samples per experiment). For the expression profiling of sample batches we followed the manufacturer’s instructions, using the 96 samples assay kit containing 8 beadchip microarrays, where each beadchip had 12 individual microarrays. In brief, 200ng of total FFPE RNA in 5μl from 48 samples in duplicate in a 96 well-plate was poly-adenylated for 1h at 37°C. The poly-adenylated miRNAs were then reverse transcribed using a biotin-labeled oligo-dT primer and the supplied reagents at 42°C for 1h. Then, a mix of miRNA-specific oligonucleotides was added and subjected to hybridization of the reverse-transcribed material for 4h on a heat-block (annealing based on temperature decrease from 70°C to 40°C). The duplex cDNA-oligonucleotides were purified by binding to magnetic streptavidin beads, and the oligonucleotides were extended to create a double-stranded DNA template and eluted from the magnetic beads. The eluted DNA products were subjected to PCR amplification (34 cycles at 95°C for 35 seconds, 56°C for 35 seconds and 72°C for 2 minutes) and purified using streptavidin magnetic beads. The individually purified PCR products (96 samples) were then loaded onto the opening of the individual microarrays (12 per beadchip) and incubated at 58°C for 16h under agitation, using the appropriate hybridization reagents. The microarrays were washed the next day using Illumina’s reagents and scanned on an Illumina beadarray reader. The raw data were extracted using GenomeStudio and exported to Excel sheets for statistical analysis.

#### Intermediate and validation stage—MiRNA reverse-transcription and quantitative PCR (qPCR)

In an intermediate stage, we selected 81 miRNAs (plus 4 controls) to be analyzed on 15 case-control pairs from the discovery stage of the study, to perform cross-platform validation between the Illumina MicroRNA profiling assay and Taqman^®^ miRNA assays and reagents (S1 Table). The 15 pairs selected for these analyses were randomly selected from the FFPE RNA specimens available, but given that a total of 81 miRNAs were analyzed at this stage and that each miRNA assay required 10 ng FFPE RNA, selection was restricted to those samples that yielded at least 1μg of RNA (75% of all samples produced at least 1μg of RNA). The correlation between data obtained from these two platforms was examined and the 14 miRNAs that were most differentially expressed between cases and controls and that also showed the strongest correlations between the two platforms were selected for the validation stage.

In the validation stage, the 14 miRNAs were each quantified in triplicate in total RNA extracted from FFPE BBD lesions of matched cases and controls using Taqman^®^ miRNA qPCR assays and reagents (Applied Biosystems, CA, USA) as described elsewhere [17]. MiR-191 and RNU6b were used as endogenous controls for data normalization [17]. Quantitative PCR measurements were performed on a Step-One-Plus qPCR instrument using the manufacturer’s recommended 96 well-plates and covers, and using the qPCR cycles recommended for Taqman^®^ miRNA quantifications. Quantitative data were then transferred to Excel sheets for statistical analysis.

### Statistical analysis

#### Discovery stage

Unconditional logistic regression was used to generate odds ratios and 95% confidence intervals (CI) for the association between lifestyle factors and breast cancer risk. Quantitative factors, including body mass index (BMI (weight (kg)/height (m^2^)), age at menarche, age at first live birth and number of pregnancies, were categorized according to their distribution in the controls. A matched pair analysis was not used because a substantial proportion of subjects had missing data on lifestyle factors. Instead, we included the matching variables, age, age at diagnosis of BBD, and duration in the cohort, as covariates in the regression model. The association between BBD pathology and breast cancer risk was analyzed using logistic regression in the same way. The categories of “No breast lesion” and “Non-proliferative lesion” were combined and treated as the reference category.

For analysis of the miRNA data from the discovery stage, obtained using the Illumina MiRNA Expression Profiling Assay, we log-transformed and quantile normalized the miRNA intensity values obtained from the Illumina bead arrays, and used them in all further analysis of miRNA expression levels. For each sample, correlation coefficients were computed between the duplicated arrays to assess repeatability. Batch effects associated with use of the Illumina arrays were discovered by visualization and by examining the statistical significance of a variable representing batch, as assessed by ANOVA. In analysis of the association between miRNA levels and breast cancer risk, a total of 246 miRNAs were excluded, as in more than 95% of the 1248 arrays they were below the detection level (P-values > 0.05 compared to background noise, as reported by Genome Studio.)

Linear regression was used to evaluate the association between the expression level of each miRNA (response) and the risk factors (explanatory variables) such as cigarette smoking, BMI, etc., using the control samples only, and adjusted for batch effect by including a factor representing batch as a covariate. In the regression, the miRNA expression level for each sample was the average of two replicates, weighted by the reciprocal of the sample variance between the two replicates.

Linear regression was also used to evaluate the association between the expression level of each miRNA and breast cancer risk, in which the average of two replicated miRNA measures was used as the response and case-control status was the explanatory variable, and the batch factor was entered as a covariate. Subjects (n = 14) with atypical hyperplasia were excluded from this analysis, as atypical hyperplasia itself was strongly associated with breast cancer risk. We computed variances between the two replicates for each sample. Those measurements with larger variances are less reliable; therefore, in the regression analysis for each miRNA, the samples were weighted based on the between-duplicate variance for that miRNA. To avoid certain samples exerting excessively high influence because of very small between-duplicate variance that might have occurred by chance, we assigned the weights in the following way: those samples whose between-duplicate variances were below the median between-duplicate variance received equal weight, at the value of the reciprocal of the median between-duplicate variance; the other half, whose between-duplicate variances were at or above the median, were weighted lower, using the reciprocal of their between-duplicate variance. From this analysis, the top 71 miRNAs with lowest P-values were selected for qPCR validation in the intermediate stage.

A similar analysis was also used to identify the miRNAs associated with atypical hyperplasia, using only the samples from cases. From this analysis, a total of 10 miRNAs with the lowest P-values were added to the intermediate stage. In further analysis, restricted to the 156 case-control pairs that showed good repeatability (r>0.95), paired t-tests were used for each miRNA to test if they were differentially expressed between two groups. We repeated the same analysis after excluding the atypical hyperplasia samples, with 149 matched case-control pairs.

#### Intermediate stage

In the intermediate stage, we used linear regression to evaluate whether the miRNA expression levels measured by the Illumina array were consistent with the expression level measured by qPCR on 15 matched case-control samples randomly selected from the 149 pairs with good repeatability. We first regressed the normalized log-intensities on the batch factor first, and took the residual. We then computed the correlation coefficient between the residuals and the qPCR measures, and obtained a P-value for statistical significance by regressing the residuals on the qPCR measure. Based on this analysis, we selected 14 miRNAs for the test stage in the following way. First, we identified 22 miRNAs for which the correlation coefficients were less than -0.25 and the P-values for correlation were below 0.05. From these, we selected the 14 miRNAs that had the greatest fold-changes between cases and controls (after adjusting for batch effect) in the discovery stage. For these 14 miRNAs, the fold-changes in the discovery stage were between 1.12 and 1.20 (in either direction).

#### Validation stage

For the analysis of miRNA data from the validation stage, obtained using qPCR, we first used linear regression to determine whether any of the breast cancer risk factors were associated with miRNA expression (average of triplicate measures for each miRNA). We then computed the average miRNA expression fold-changes between the cases and controls and performed paired t-tests on the 165 matched case-control pairs included in the validation stage. In addition, we also tested for case-control differences by using linear regression models on the 318 samples in which miRNA expression (the average of the triplicate measures) was the response and the final disease status of the patient and the assay plates were the independent variables. Other independent variables in the models were the three variables used to match the samples, namely age at BBD diagnosis, age, and duration in the cohort.

All statistical tests were two-sided and all data analyses were performed using R (www.r-project.org).

## Results

S1 Fig shows the number of study subjects by study stage. The discovery stage included 704 subjects, of whom 692 had chart data, and of whom 608 also had sufficient tissue for miRNA analysis. For the validation stage, the corresponding figures were 378, 365 and 314, respectively.

### Discovery stage

#### Association between lifestyle factors, benign breast disease histology, and breast cancer risk

In the discovery stage of the study, chart data were obtained for 353 cases and 339 controls (constituting 353 case-control pairs because some controls were matched to more than one case). Table 1 shows the association between various factors and risk of invasive breast cancer subsequent to BBD (the associations for the entire cohort have been reported elsewhere [18]). Most of the factors examined were not associated with altered risk. However, there was some suggestion of a reduction in risk of breast cancer in association with a bilateral oophorectomy, and there was a statistically significant 4-fold increase in risk in women who had ever used hormone therapy. Additionally, for the 689 participants that had pathology data, there was a strong positive association between atypical hyperplasia and risk of subsequent breast cancer (Table 2).

**Table 1:** Association between lifestyle factors and breast cancer risk based on subjects included in the discovery stage.

Variable	Level	No. of cases (N = 353)	No. of controls (N = 339)	OR (95% CI)
Ever smoked cigarettes	No	120	123	1^a^
	Yes	139	154	0.93 (0.66,1.30)
	Missing	94	62	
BMI (kg/m^2^)	< 23.38	107	103	1^a^
	23.38–27.92	104	106	0.94 (0.64,1.38)
	>27.92	108	101	1.03 (0.71,1.50)
	Missing	34	29	
	*P trend*			0.95
Age at menarche (years)	≤11	56	58	1^a^
	12–13	154	165	0.97 (0.63,1.48)
	≥14	72	66	1.13 (0.69,1.86)
	Missing	71	50	
	*P trend*			0.83
Age at first live birth (years)	Never had	50	40	1^a^
	15–19	47	46	0.82 (0.46,1.46)
	20–24	118	129	0.73 (0.45,1.19)
	25–29	47	65	0.58 (0.33,1.01)
	≥30	35	22	1.27 (0.65,2.5)
	Missing	56	37	
	*P trend*			0.68 ^b^
Number of pregnancies	Never pregnant	47	37	1^a^
	1	38	49	0.61 (0.33,1.12)
	2	113	118	0.75 (0.46,1.25)
	3	68	66	0.81 (0.47,1.4)
	≥4	65	57	0.90 (0.51,1.57)
	Missing^c^	18	10	
	*P trend*			0.42
Menopausal status	Premenopausal	141	130	1^a^
	Postmenopausal	140	162	0.80 (0.57,1,11)
	Missing	71	34	
History of bilateral oophorectomy	No	274	256	1^a^
	Yes	36	52	0.65 (0.41, 1.02)
	Missing	43	31	
History of breast cancer in first degree relative	No	268	274	1^a^
	Yes	62	45	1.32 (0.86, 2.00)
	Missing	23	20	
Ever used hormone therapy	No	8	37	1^a^
	Yes	159	179	4.11 (1.86,9.08)
	Missing	186	123	

^a^ Reference category

^b^ Subjects that had never had a pregnancy are excluded from the test for trend

^c^4 cases, 2 controls, had been pregnant but had not completed their pregnancy; 1 case, 13 controls were perimenopausal.

**Table 2:** Association between benign breast disease histology and breast cancer risk.

Histologic category	No. of cases	No. of controls	OR (95% CI)
No lesion or non-proliferative lesion^a^	101	98	1^b^
Epithelial hyperplasia without atypia	236	238	0.96 (0.69,1.34)
Atypical hyperplasia	14	2	6.79 (1.50, 30.67)

^a^14 cases, 13 controls had no breast lesion.

^b^ Reference category.

#### miRNA assays

All miRNA assays in the discovery stage were done in duplicate. We evaluated the 618 samples (316 case-control pairs) that were processed, and computed the correlation coefficients between the two replicates. The median was 0.973, and 388 (about 63%) of the samples had correlations greater than 0.95. We evaluated the p-values reported by Genome Studio for detection of each miRNA on 1248 arrays. A total of 246 miRNAs were below the detection level (P-value < 0.05) in more than 60 arrays. We excluded these 246 miRNAs in assessing the false discovery rate. We also evaluated the relation between cigarette smoking, BMI, and miRNA expression among the control samples. There were no statistically significant associations.

There was a very strong batch effect in the miRNA assays. S2 Fig illustrates the batch effects by plotting the log-intensity of miRNA HS_10 and miRNA HS_104 for all 618 samples against the order in which they were processed. Each batch had 48 samples. Thirteen batches are separated by the vertical lines in the plot. The top plot shows the result of the first replicate of each sample, and the bottom one shows the results of the second replicate. Given this observation, we corrected for the batch effect of miRNA expression by including batch as a factor in the regression models relating miRNA to breast cancer risk.

#### miRNA expression and breast cancer risk

First, for each miRNA, we weighted each sample based on the sample variance between the two replicate measurements for that miRNA. While the samples showing low variance (below the median for all samples) received the same weight, the samples with high variance (at or above the median for all samples) received lower weight (proportional to the reciprocal of the variance). The results showed no signal, and excluding the atypical hyperplasia samples did not improve the signal. Second, we weighted each sample by their repeatability measure (r^2^)—the samples with r>0.973 received equal weights. This weighting is very light, as the lowest weight is about ⅓ of the highest weight. Among 899 miRNAs that were above the detection level, a total of 114 miRNA were associated with risk of breast cancer at P-values < 0.05 (FDR = 0.40), 79 were associated with risk at P-values < 0.025 (FDR = 0.28), and 47 were associated with risk at P-values < 0.01 (FDR = 0.20). We selected the 71 miRNAs with the smallest P-values for PCR validation.

#### miRNA association with atypical hyperplasia

We investigated if any miRNAs showed differences in expression levels between atypical hyperplasia and other samples. We limited our analysis to the cases, and compared the 12 atypical hyperplasia samples with the 304 in the other pathology categories. We selected 10 additional miRNAs with the lowest P-values that were not already in the list of 71 miRNA selected from the case-control analysis (see above) for PCR validation.

### Intermediate stage

From the 81 candidate miRNAs (see list in S1 Table), we selected the following 14 miRNAs for inclusion in the validation stage of the study based on their expression levels and the size and the statistical significance of the correlation between the results of the Illumina array and the qPCR results (in 15 case-control pairs from the discovery stage): miR-193b, miR-10b, miR-503, miR-1247, miR-425, miR-500*, miR-502-3p, miR-150, miR-383, miR-376b, miR-33b, miR-7, miR-501-5p, and miR-4791. In a sensitivity analysis, we performed the same analysis on the discovery stage data but only used the cases for which the breast cancer and the BBD occurred in the same breast (a total of 178), and we found 11 of the selected 14 miRNAs were significantly associated with risk (P-value < 0.1)(data not shown).

### Validation stage

In the validation stage of the study, chart data were obtained for 190 cases and 175 controls (constituting 190 case-control pairs because some controls were matched to more than one case). The risk factor associations with breast cancer observed in the validation stage were broadly similar to those observed in the discovery stage (S2 Table). There were no associations between the breast cancer risk factors listed in the table and expression of the 14 miRNAs assessed in the validation stage (as well as the 2 miRNAs that served as controls)(data not shown). Furthermore, there were no statistically significant differences between the cases and matched controls in expression of these 14 miRNAs. The average fold-change, the P-values and observed test statistics of both the paired t-tests and from the linear regression models are shown in Table 3. We also repeated the linear regression analysis but excluded the cases in which the BBD and breast cancer occurred in different breasts. With the remaining 86 cases and 150 controls, we found no statistically significant associations.

**Table 3:** Fold changes and p-values for the 14 miRNAs (plus 2 controls) evaluated in the validation stage.

	miRNA	Average miRNA expression fold change: cases vs controls	T-stat df = 151	Pval Paired T	F-Stat df = 1,158	Pval Linear
1	miR-193b	0.97	0.396	0.69	0.001	0.97
2	miR-10b	1.00	-0.061	0.95	0.005	0.94
3	miR-503	1.06	-0.625	0.53	0.217	0.64
4	miR-1247	1.03	-0.441	0.67	0.296	0.59
5	miR-4791	0.97	0.576	0.56	0.133	0.72
6	miR-425	0.90	1.525	0.13	1.224	0.27
7	miR-500	1.10	-1.293	0.20	2.048	0.15
8	miR-502-3p	1.06	-0.962	0.34	1.496	0.22
9	miR-150	0.96	-0.442	0.66	0.272	0.60
10	miR-383	0.98	-0.346	0.73	0.149	0.70
11	miR-376b	1.02	-0.223	0.82	0.001	0.97
12	miR-33b	0.92	1.154	0.25	1.516	0.22
13	miR-7	0.90	1.372	0.17	1.658	0.20
14	miR-501-5p	1.04	-0.722	0.47	0.634	0.43
15	miR-19(as control)	0.94	1.105	0.27	0.721	0.40
16	RNU6B(as control)	1.05	-0.667	0.51	2.577	0.11

## Discussion

MiRNAs are master regulators of gene expression that bind to imperfect complementary sites within the 3’ untranslated regions of their mRNA targets and direct mRNA degradation and/or posttranscriptional repression [6]. Dysregulation of miRNA expression contributes to the hallmarks or traits of cancer. These traits, including sustaining proliferative signalling, evading growth suppressors, resisting cell death, enabling replicative immortality, and evading immune destruction, are distinctive and complementary capabilities that enable tumor growth [19–21]. Abnormal expression of miRNAs is thought to contribute to the development and progression of many cancers, including breast cancer [4–7].

The results of the study reported here, which included both discovery and validation stages, suggest that, in contrast to the putative role of miRNAs in breast cancer metastasis [7], miRNA expression in benign breast tissue is not related to the risk of subsequent incident, invasive breast cancer. Although we are not aware of any previous large-scale prospective studies that have examined the association between miRNA expression and breast cancer risk, in a recent small, multi-stage study, the validation stage showed that expression of miR-18a and miR-210 was significantly higher in benign breast biopsy samples from 40 women who subsequently developed invasive breast cancer than in benign biopsy samples from 40 women who did not develop subsequent invasive breast cancer [22]. However, the underlying cohort was not defined, and the results were not adjusted for potential confounding factors (e.g., hormone use).

The present study has several strengths. First, it was conducted in a large cohort of women with BBD that was well characterized with respect to the histology of the benign lesions (based on standardized review of each histologic section) and risk factor information, with long-term follow-up for breast cancer incidence. Second, we used a standardized chart abstraction form to obtain data from the KPNW medical records, which have been shown to be of high quality [23,24]. Although data were missing for some variables, the proportion of missing values was generally relatively low (with the exception of ever use of hormone therapy). Third, although now discontinued (and largely replaced by sequencing), the Illumina multiplexed assay was the state-of-the-art approach at the time that the first stage of the study was conducted and it allowed the sensitive detection of miRNAs in the FFPE tissue. Fourth, for validation and single transcript analysis, we used quantitative PCR, which is currently considered to be the gold standard for determining miRNA expression levels. Fifth, to address the potential for assay variability, the laboratory technicians underwent intensive training in the study methods prior to each stage of the project. Furthermore, we employed strict quality control for RNA extraction, preparation, and quantification, and built in controls to monitor the accurate performance of our assays. Sixth, we performed the Illumina assays in duplicate (on separate 96-well plates) and the qPCR assays in triplicate.

The study also had several potential limitations. First, tissue was not available for all selected subjects. However, miRNA profiles are unlikely to have differed between subjects for whom tissue was unavailable/unsuitable for analysis and those for whom it was available/suitable. Second, RNA obtained from FFPE tissue is often degraded. However, we have demonstrated considerable success in identifying and characterizing miRNA expression patterns using RNA extracted from FFPE tissue and in imposing strict quality control measures, and we have shown excellent agreement between miRNA expression levels in fresh and matched FFPE tissue [10]. Third, there was a strong batch effect in the discovery stage assays, possibly due to the fact that the miRNA bead array analyses were performed over a period of 2 years, during which different batches of reagents and arrays were acquired. Nevertheless, we corrected for this by including batch as a factor in the regression models relating miRNA expression to breast cancer risk. Fourth, although it is conceivable that the laboratory assays resulted in some individuals being misclassified with respect to their miRNA expression profile, the methods that we used are highly sensitive and repeatable and (as indicated earlier) were performed in duplicate (Illumina multiplexed assay) for the discovery stage and in triplicate (qRT-PCR) for the validation stage.

In conclusion, this study, which included both discovery and validation components, did not show that dysregulated expression of miRNAs in benign breast disease tissue is associated with altered risk of subsequent invasive breast cancer. However, in place of array-based methods, use of a more sensitive and high-throughput technology, such as sequencing [25], may be needed to rule out a role for miRNAs in the development of breast cancer.

## Supporting information

S1 Fig Supplementary Figure 1. Flow chart showing study subject inclusion by stage of study. (1112 Records (1082 Unique Blocks/Participants)); *indicates additional samples lost due to elimination of the matched pair.
(DOCX)

S2 Fig Supplementary Figure 2. Batch effects for miRNA HS_10 and miRNA HS_104.
(DOCX)

S1 Table Supplementary Table 1. Candidate miRNAs from which the 14 miRNAs included in the validation case-control study were selected.
(DOCX)

S2 Table Supplementary Table 2. Association between lifestyle factors and breast cancer risk based on subjects included in the validation stage.
(DOCX)
