# Supplementary material for: MicroRNA expression in benign breast tissue and risk of subsequent invasive breast cancer
Source: PLoS One. 2018 Feb 12;13(2):e0191814. doi: 10.1371/journal.pone.0191814 (PMC5809016; doi:10.1371/journal.pone.0191814)
Supplement: S1 Fig — Flow chart showing study subject inclusion by stage of study. (1112 Records (1082 Unique Blocks/Participants)); *indicates additional samples lost due to elimination of the matched pair. (DOCX) [file pone.0191814.s001.docx]

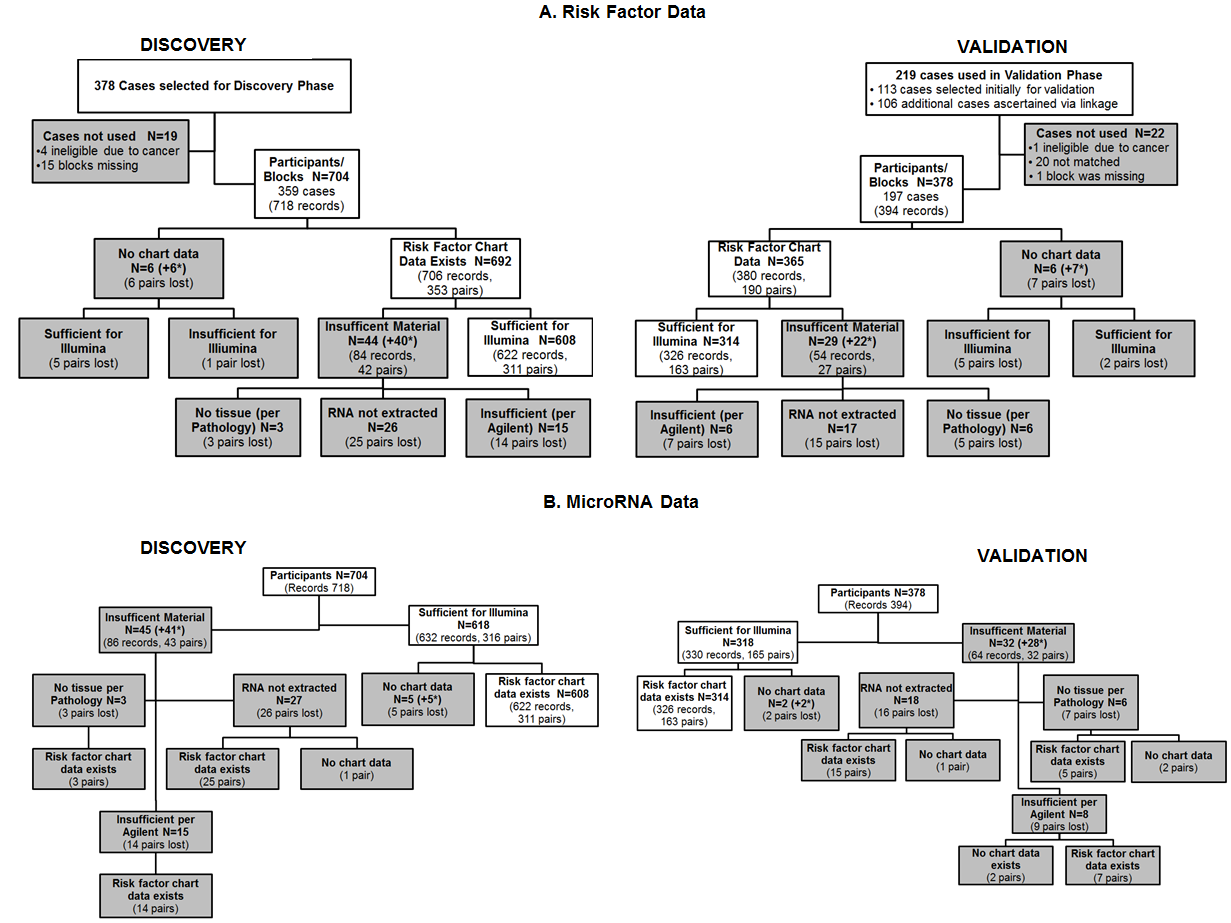


**Supplementary figure 1.** Flow chart showing study subject inclusion by stage of study. (1112 Records (1082 Unique Blocks/Participants)); *indicates additional samples lost due to elimination of the matched pair.

**CHART DATA-DISCOVERY STAGE:**

**CONTROLS:** 332 participants with 1 control record, 6 participants with 2 controls records,1 participant with 3 control records, 6 participants whose records were used as control and a case=353 control records. **CASES:** 347 participants with one case record, 6 participants who also had a control record=TOTAL CASE RECORDS=353

**CHART DATA-VALIDATION STAGE:**

**CONTROLS:** 168 participants with 1 control record, 7 participants with 2 controls records, 8 participants whose records were used as control and a case=190 control records. **CASES:** 182 participants with one case record, 8 participants who also had a control record=TOTAL CASE RECORDS=190

**CHART DATA-ALL:**

**CONTROLS:** 500 participants with 1 control record,13 participants with 2 controls records,1 participant with 3 control records , 14 participants whose records were used as control and a case= 543 control records. **CASES:** 529 participants with one case record, 14 participants who also had a control record=TOTAL CASE RECORDS=543.
