# Supplementary material for: MicroRNA expression in benign breast tissue and risk of subsequent invasive breast cancer
Source: PLoS One. 2018 Feb 12;13(2):e0191814. doi: 10.1371/journal.pone.0191814 (PMC5809016; doi:10.1371/journal.pone.0191814)
Supplement: S2 Fig — Batch effects for miRNA HS_10 and miRNA HS_104. (DOCX) [file pone.0191814.s002.docx]

**Supplementary figure 2.** Batch effects for miRNA HS_10 and miRNA HS_104.
