# Supplementary material for: MicroRNA expression in benign breast tissue and risk of subsequent invasive breast cancer
Source: PLoS One. 2018 Feb 12;13(2):e0191814. doi: 10.1371/journal.pone.0191814 (PMC5809016; doi:10.1371/journal.pone.0191814)
Supplement: S1 Table — Candidate miRNAs from which the 14 miRNAs included in the validation case-control study were selected. (DOCX) [file pone.0191814.s003.docx]

**Supplementary Table 1.** Candidate miRNAs from which the 14 miRNAs included in the validation case-control study were selected.

Controls: RNU44 RNU6b RNU48 Mir-202

Tested miRNAs:

| Hsa-mir-96 | Hsa-mir-503 | HS_257 | Hsa-mir-664 |
| --- | --- | --- | --- |
| Hsa-mir-182 | Hsa-mir-4791 | HS_260 | Hsa-mir-29b-2* |
| Hsa-mir-632 | HS_81 | HS_263.1 | Hsa-mir-10b |
| Hsa-mir-501-3p | Hsa-mir-150 | Hsa-mir-30a* | Hsa-mir-425 |
| Hsa-mir-595 | Hsa-mir-193b | Hsa-mir-191 | Hsa-mir-30c-1* |
| Hsa-mir-224 | Hsa-mir-502-3p | Hsa-mir-3940-3p | Hsa-mir-500* |
| Hsa-mir-331-3p | Hsa-mir-574-5p | Hsa-mir-222 | Hsa-mir-130a-5p |
| Hsa-mir-625 | Hsa-mir-1247 | Hsa-mir-513a-5p | Hsa-mir-1293 |
| Hsa-mir-483-3p | Hsa-mir-3187 | Hsa-mir-302a | Hsa-mir-6670-5p |
| Hsa-mir-551b | RNU-102 | Hsa-mir-1282 | Hsa-mir-3912 |
| Hsa-mir-376b | Hsa-mir-125b | Hsa-mir-1307 | HS_203 |
| Hsa-mir-346 | Hsa-mir-501-5p | Hsa-mir-512-5p | Hsa-mir-624 |
| Hsa-mir-29b | Hsa-mir-1274a | Hsa-mir-33b | Hsa-mir-155 |
| Hsa-mir-365 | Hsa-mir-517c | Hsa-mir-1183 | Hsa-mir-127-5p |
| Hsa-mir-335 | HS_4.1 | Hsa-mir-661 | Hsa-mir-1181 |
| Hsa-mir-135b | Hsa-mir-34a | Hsa-mir-7 | Hsa-mir-181c-3p |
| Hsa-mir-7-1* | Hsa-mir-217 | Hsa-mir-18a | Hsa-mir-1197 |
| Hsa-mir-1303 | Hsa-mir-522 | Hsa-mir-539 | Hsa-mir-375 |
| Hsa-let-7g | Hsa-mir-383-5p | Hsa-mir-139-5p | Hsa-mir-603 |
| Hsa-mir-1179 | Hsa-mir-593 | Hsa-mir-144-5p | Hsa-mir-191 |
|  |  |  | Hsa-mir-518f |
