# Supplementary material for: MicroRNA expression in benign breast tissue and risk of subsequent invasive breast cancer
Source: PLoS One. 2018 Feb 12;13(2):e0191814. doi: 10.1371/journal.pone.0191814 (PMC5809016; doi:10.1371/journal.pone.0191814)
Supplement: S2 Table — Association between lifestyle factors and breast cancer risk based on subjects included in the validation stage. (DOCX) [file pone.0191814.s004.docx]

**Supplementary Table 2.** Association between lifestyle factors and breast cancer risk based on

subjects included in the validation stage.

| **Variable** | **Level** | **No. of cases (N=190)** | **No. of controls (N=175)** | **OR (95% CI)** |
| --- | --- | --- | --- | --- |
| Ever smoked cigarettes | No | 78 | 73 | 1* |
|  | Yes | 72 | 75 | 0.90 (0.57,1.42) |
|  | Missing | 40 | 27 |  |
|  |  |  |  |  |
| BMI (kg/m^2^) | < 23.38 | 57 | 47 | 1* |
|  | 23.38 - 27.92 | 50 | 49 | 0.84 (0.48,1.46) |
|  | >27.92 | 62 | 61 | 0.84 (0.50,1.41) |
|  | Missing | 21 | 18 |  |
|  | *P trend* |  |  | 0.152 |
|  |  |  |  |  |
| Age at menarche (years) | ≤11 | 26 | 19 | 1* |
|  | 12-13 | 76 | 70 | 0.79 (0.40,1.56) |
|  | ≥14 | 35 | 31 | 0.83 (0.38,1.77) |
|  | Missing | 53 | 55 |  |
|  | *P trend* |  |  | 0.578 |
|  |  |  |  |  |
| Age at first live birth (years) | Never had | 28 | 20 | 1* |
|  | 15-19 | 19 | 16 | 0.85 (0.35,2.04) |
|  | 20-24 | 54 | 56 | 0.69 (0.35,1.37) |
|  | 25-29 | 25 | 31 | 0.57 (0.26,1.26) |
|  | ≥30 | 22 | 15 | 1.05 (0.44,2.50) |
|  | Missing | 42 | 37 |  |
|  | *P trend* |  |  | 0.532 |
|  |  |  |  |  |
| Number of pregnancies | Never pregnant | 27 | 20 | 1* |
|  | 1 | 20 | 26 | 0.57 (0.25,1.30) |
|  | 2 | 62 | 58 | 0.79 (0.40,1.56) |
|  | 3 | 39 | 33 | 0.88 (0.42,1.83) |
|  | ≥4 | 28 | 23 | 0.90 (0.41,2.01) |
|  | Missing | 14 | 15 |  |
|  | *P trend* |  |  | 0.475 |
|  |  |  |  |  |
| Menopausal status | Premenopausal | 74 | 55 | 1* |
|  | Postmenopausal | 83 | 82 | 0.75 (0.47,1,20) |
|  | Missing | 27 | 29 |  |
|  |  |  |  |  |
| History of bilateral oophorectomy | No | 157 | 136 | 1* |
|  | Yes | 21 | 27 | 0.67 (0.36, 1.25) |
|  | Missing | 12 | 12 |  |
|  |  |  |  |  |
| History of breast cancer in first degree relative | No | 140 | 133 | 1* |
|  | Yes | 36 | 27 | 1.27 (0.73, 2.20) |
|  | Missing | 14 | 15 |  |
|  |  |  |  |  |
| Ever used hormone therapy | No | 4 | 18 | 1* |
|  | Yes | 93 | 88 | 4.76 (1.55,14.6) |
|  | Missing | 93 | 69 |  |
|  |  |  |  |  |
